# Supplementary material for: Reading ability and phoneme categorization
Source: Sci Rep. 2018 Nov 15;8:16842. doi: 10.1038/s41598-018-34823-8 (PMC6237901; doi:10.1038/s41598-018-34823-8)
Supplement: Supplementary file 1 — Supplementary Materials [file 41598_2018_34823_MOESM1_ESM.pdf]

Reading ability and phoneme categorization

Gabrielle E. O'Brien, Daniel R. McCloy, Emily C. Kubota, and Jason D. Yeatman
Institute for Learning and Brain Sciences and Department of Speech and Hearing Sciences, University of Washington, Seattle, WA, USA

Supplementary Materials

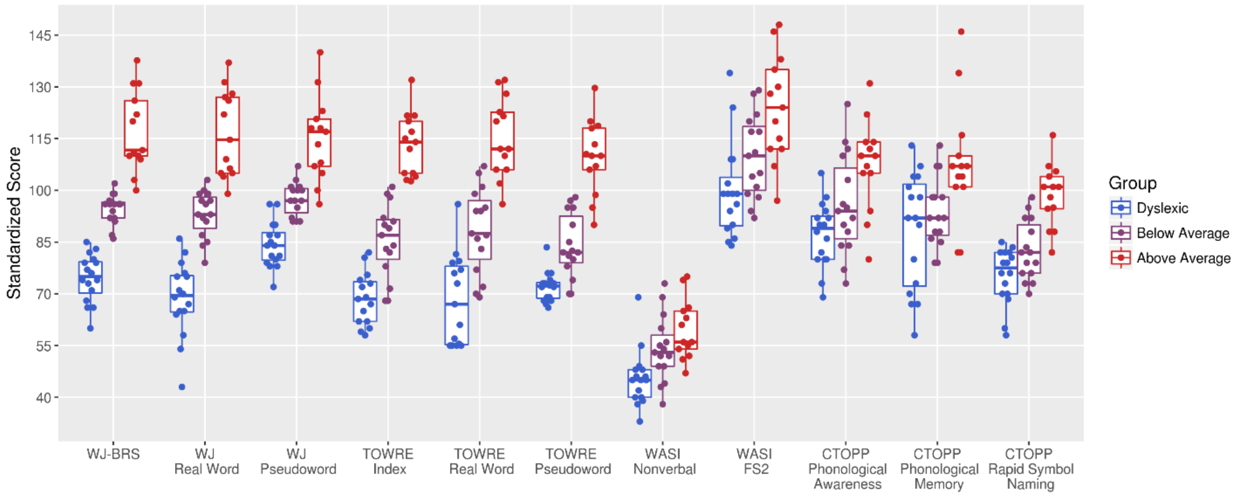

Figure S1: *Demographic information for all 44 participants on the study on various behavioral measures, including the Woodcock-Johnson IV (WJ), Test of Word Reading Efficiency (TOWRE), Weschler Abbreviated Scale of Intelligence (WASI), and the Comprehensive Test of Phonological Processing (CTOPP). Each dot represents an individual subject, box plots denote the 25th, 50th, and 75th percentiles, and color marks the group assigned to each subject. Note that all standardized scores are on the same scale (μ = 100) except the WASI Nonverbal measure (μ = 50).*

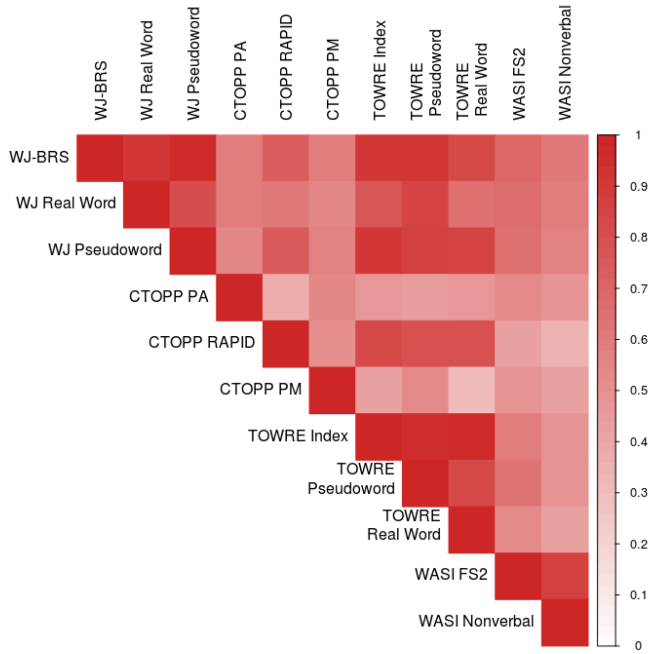

Figure S2: *Correlation plot for behavioral measures relating to reading, phonological processing, and cognitive abilities. All correlations are highly significant after correction for multiple comparisons (p < 1e-8).*

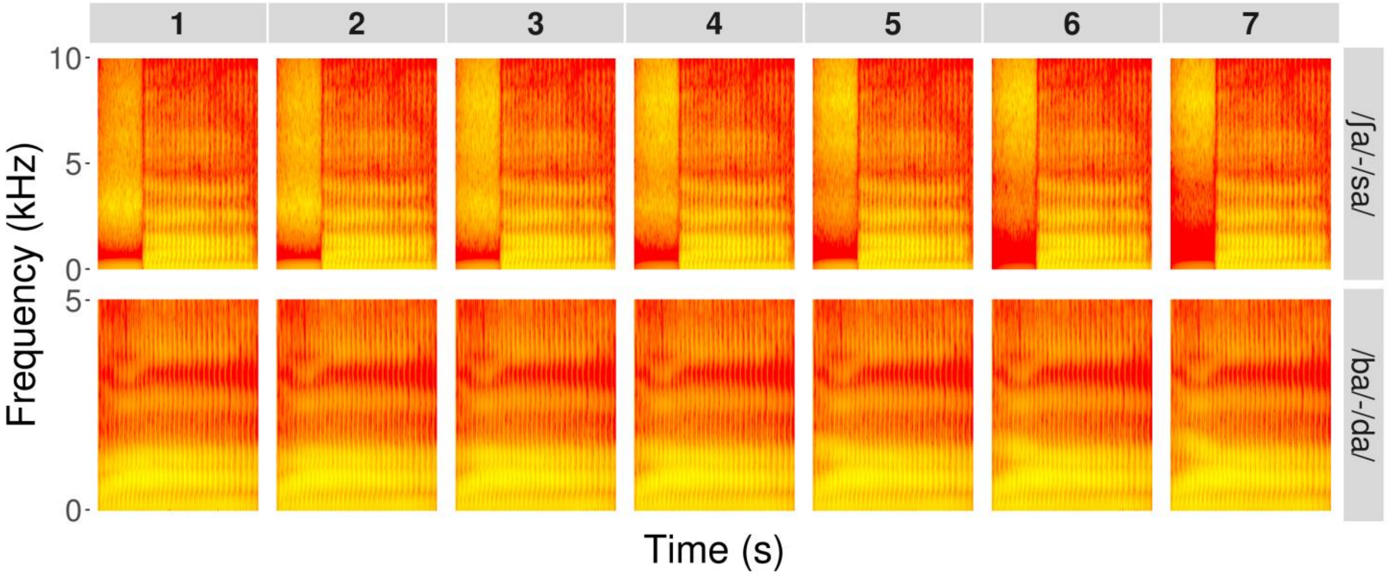

Figure S3: *Spectrograms of the endpoints of the two speech continua. Top row: the endpoints of the /sa/-/ʃa/ continuum, which differ based on the relative amplitudes of spectral peaks in the initial fricative. Bottom row: the endpoints of the /ba/-/da/ continuum, which differ based on starting frequency of the initial F2 transition. Note the different y-axis scales in the top and bottom rows.*

Stepwise model selection procedure

Fixed-effect predictors with sum coding were used for both the continuum (static /ʃa/~/sa/ versus dynamic /ba/~/da/) and paradigm (ABX versus single-stimulus) variables. Reading ability (WJ-BRS score) was entered as a continuous fixed-effect predictor except where otherwise stated. Nuisance predictors were added for presence/absence of ADHD diagnosis (treatment coding) and for non-verbal IQ (WASI-III Matrix Reasoning; continuous predictor). A random effect for participant was also included.

Model of psychometric slope

The fully-specified model of psychometric slope indicated that only one predictor—WJ-BRS reading score—showed a significant effect on slope estimates. To refine this model, first the nuisance parameters (ADHD diagnosis and non-verbal IQ) were each removed, and those subset models were tested against the full model using likelihood ratio tests and a conservative threshold for parameter exclusion of $p > 0.1$. The tests indicated no improvement in model fit from the inclusion of the nuisance predictors. Next, the predictors for continuum and paradigm (and their interactions with each other and with reading ability) were removed from the reduced model. Again, likelihood ratio tests indicated no significant improvement of model fit from the inclusion of those predictors, suggesting that the most parsimonious model of psychometric slope contained only a continuous predictor of reading ability (WJ-BRS) and a random intercept for each participant.

Model of lapse rate

Following the same procedure as above, both nuisance predictors (ADHD diagnosis and non-verbal IQ) were eliminated, as were the predictor for continuum type and its interactions with reading ability and paradigm, and the interaction between reading ability and continuum. This yielded a final model of lapse rate containing a continuous predictor for reading ability, a categorical predictor for continuum, and a random effect for participant.

Quadratic and Linear Discriminant Analysis

To assess whether individuals in the Dyslexic group could be identified as such based on their psychometric functions, we first computed each subject's average psychometric function by averaging the slope and two asymptote parameters across all six blocks of the experiment. Using a leave-one-out approach, a quadratic discriminant analysis (or linear discriminant analysis, where noted) of group identity using slope and the average lapse rate was performed on a training set comprised

of all data except the held-out point. Then the class of the held-out point was predicted. This was repeated holding out each subject's average psychometric function, and in this way every subject could be assigned a label without overfitting.
